# Oxygenation performance assessment of an artificial lung in different central anatomic configurations

**DOI:** 10.1177/03913988231168163

**Published:** 2023-04-12

**Authors:** Flutura Hima, Sebastian Kalverkamp, Ali Kashefi, Khosrow Mottaghy, Rachad Zayat, Lasse Strudthoff, Jan Spillner, Foivos Mouzakis

**Affiliations:** 1 Clinic for Thoracic Surgery, University Hospital RWTH Aachen, Aachen, Germany; 2 Institute of Physiology, RWTH Aachen University, Aachen, Germany; 3 Department of Cardiovascular Engineering, Institute of Applied Medical Engineering, RWTH Aachen University, Aachen, Germany

**Keywords:** Gas exchange, central anatomic configuration, mock loop, numerical simulation, artificial lung and respiratory support

## Abstract

**Objectives:**

Aim of this work was to characterize possible central anatomical configurations in which a future artificial lung (AL) could be connected, in terms of oxygenation performance.

**Methods:**

Pulmonary and systemic circulations were simulated using a numerical and an in vitro approach. The in vitro simulation was carried out in a mock loop in three phases: (1) normal lung, (2) pulmonary shunt (50% and 100%), and (3) oxygenator support in three anatomical configurations: right atrium-pulmonary artery (RA-PA), pulmonary artery-left atrium (PA-LA), and aorta-left atrium (Ao-LA). The numerical simulation was performed for the oxygenator support phase. The oxygen saturation (SO_2_) of the arterial blood was plotted over time for two percentages of pulmonary shunt and three blood flow rates through the oxygenator.

**Results:**

During the pulmonary shunt phase, SO_2_ reached a steady state value (of 68% for a 50% shunt and of nearly 0% for a 100% shunt) 20 min after the shunt was set. During the oxygenator support phase, physiological values of SO_2_ were reached for RA-PA and PA-LA, in case of a 50% pulmonary shunt. For the same conditions, Ao-LA could reach a maximum SO_2_ of nearly 60%. Numerical results were congruous to the in vitro simulation ones.

**Conclusions:**

Both in vitro and numerical simulations were able to properly characterize oxygenation properties of a future AL depending on its placement. Different anatomical configurations perform differently in terms of oxygenation. Right to right and right to left connections perform better than left to left ones.

## Introduction

Lung diseases are the third most frequent causes of death nowadays. To provide respiratory support to patients suffering from lung diseases, extracorporeal membrane oxygenation (ECMO) can be used as bridge therapy (to recovery or transplant) in selected cases.^1^ A long term solution such as an artificial lung (AL), as well as its properties and requirements are still in a conceptual phase.^2^

While some working groups focus on improvement of wearable devices such as MLung,^3^ and PAL,^4^ others are concentrated on investigating the best placement for a future AL. Different anatomical positions of a possible connection of an AL may influence gas exchange properties. Steuer et al.^5^ aimed to create a venovenous connection to the common iliac veins. A peripheral cannulation was used also by Reng et al.^6^ for their pumpless extracorporeal lung assist.

According to our assessment, a central cannulation in or near the heart will most likely be more suitable for a future AL because it allows generation of high blood flows. Although the choice of one configuration or another may seem intuitive, it highly depends on the need of the patient (e.g., pulmonary hypertension) and the choice is a trade-off between the advantages and disadvantages of each. There are several possible configurations to centrally connect an AL: right to right, right to left, and left to left ones. Each configuration implies different physiologies in terms of pump needed, gas- and blood flows, and potential advantages and disadvantages. However, still no data about physiological- and gas exchange properties in different anatomical configurations have been published.

The aim of this work is the numerical and in vitro characterization of the oxygenation properties in different theoretical central anatomical configurations over time for two percentages of pulmonary shunt. Taking these properties into account, the physiological potentials of each configuration are discussed.

## Materials and methods

### Anatomical configurations and basic considerations

For each central configuration considered, the possibility to generate a passive flow or the necessity of a pump was evaluated. A passive flow is generated if the pressure difference between the two sites connected is high enough to overcome the resistance of the components constituting the configuration such as membrane oxygenators.

**Figure 1.**
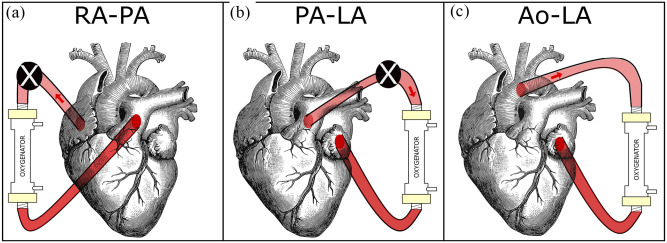
Three possible configurations for the connection of a future artificial lung (AL). A pump (represented by the black circle with superimposed white X) is needed for the RA-PA configuration and for high flows in the PA-LA configuration: (a) RA-PA, (b) PA-LA, and (c) Ao-LA.

Basically, an AL may be connected in the configuration right atrium-pulmonary artery (RA-PA). This configuration comes closest to a venovenous ECMO (VV ECMO). The pressure in the right atrium (RA) is lower than the one in the pulmonary artery (PA). Therefore, in this configuration, because of the negative pressure difference between RA and PA, a passive flow in not possible and a pump would be necessary (Figure 1(a)). Since the blood coming from this configuration flows in the lungs as well, this configuration is placed in series with the lungs and as such is the only connection that can perfuse the lungs with oxygenated blood.

A possible configuration, pulmonary artery-left atrium (PA-LA), is similar to a venoarterial ECMO (VA ECMO) because it decompresses the load from the pulmonary circulation. This configuration is in parallel to the lungs since it bypasses the lungs and as a consequence does not perfuse them with oxygenated blood. There is a physiological pressure difference between PA and left atrium (LA) which theoretically allows a passive flow. However if a membrane oxygenator is connected between the two sites, its resistance should be considered to calculate the maximum passive flow generated. A low-resistance membrane oxygenator (iLA membrane oxygenator), for instance, has a resistance of 5 mmHg/[L/min], which is five times that of a native lung.^7^ According to Spillner et al.^8^ if an iLA is connected between PA and LA, a passive flow up to 2.6 L/min is generated. For higher flows, a pump would be needed (Figure 1(b)). Even though this configuration may seem the most appropriate, it presents surgical difficulties. The cannulation of the LA is challenging and can introduce the possibility of a thrombus in the LA and as such the risk of systemic embolization.

Another theoretical connection may be between Aorta (Ao) and LA (Ao-LA).^9^ It roughly corresponds to a peripheral interventional lung assist (iLA) as used primarily to remove carbon dioxide (CO_2_). The pressure difference between Ao and LA is physiologically so high that allows even higher flow rates without the need of a pump (pumpless flow). Since the position where the oxygenated blood returns to the body (LA) is before the position from where the blood is withdrawn, this configuration causes relevant recirculation of the blood (Figure 1(c)). Although the least intuitive of the three, it is important to considerate this configuration for comparison not only as exemplar of the left-left connection but also for the advantage of generating high flows without the necessity of a mechanical pump.

This work consisted of two parts: an in vitro simulation with porcine blood and a numerical simulation performed to verify the results from in vitro. The aim was to simulate the contribution of an oxygenator connected in the three above mentioned configurations for a defined percentage of pulmonary shunt and different blood flows through the oxygenator. However, to get a complete overview of the consequences of a pulmonary shunt and to define the boundary conditions for the numerical simulation, the in vitro simulation was extended to the two phases prior to the oxygenator support one: normal lung functioning and pulmonary shunt without support. The pulmonary shunt was simulated by an oxygenator in parallel with a shunt where the blood would not get oxygenated. The percentage of the blood flow not oxygenated corresponds to the percentage of the pulmonary shunt.

We decided to focus solely on the oxygenation capabilities of the three possible central cannulations. Therefore, both simulations were performed regarding oxygenation.

### In vitro simulation

**Figure 2.**
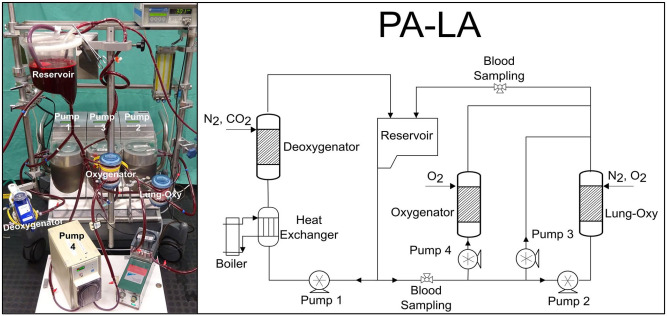
The in vitro circuit used during the experiments (left) and a schematic representation of the circuit (right). The oxygenator is connected in the PA-LA configuration.

The pulmonary and systemic circulation were simulated in a mock loop using two separate subcircuits which consist of four roller pumps (3 S3, Stockert, Germany, and 1 custom made), three commercially available oxygenators, polyvinyl chloride (PVC) tubes with an inside diameter of 3/8 in, and a reservoir (Figure 2).

The blood flow rate was measured through a clamp-on transonic flow sensor, connected to a T110 Laboratory Tubing Research flowmeter (Transonic Systems, Inc., Ithaca, USA). The circuit containing only one deoxygenator was best suited for low blood flows (human physiological flows would require more modules of deoxygenators and would make the circuit more complex). For practical reasons, a downscaling of 1/10 was performed. In the first subcircuit, the blood coming from the reservoir goes through a deoxygenator (Quadrox PLS-i, Maquet, Rastatt, Germany), which eliminates oxygen (O_2_) and increases CO_2_ in blood through a mixture of nitrogen (N_2_) (4 L/min) and CO_2_ (1 L/min). The blood is then pumped back into the reservoir. This part represents the systemic circulation. The second part of the circulation differs depending on the three phases of our experiment.

(1) During normal lung function, 600 mL/min blood, the total blood flow considered, was pumped through an oxygenator (lung-oxy) (multiECCO2R, Euroset, Medolla, Italy), where a mixture of O_2_ and N_2_ is applied to reach physiological arterial saturation (SO_2_) (98 (±2) %).
(2) During pulmonary shunt, part of the total blood flow was pumped through a bypass where it does not get oxygenated. Two percentages of pulmonary shunt were investigated: 50% and 100%. In case of a 50% pulmonary shunt, 50% of the blood flowed through the lung-oxy where it got oxygenated and then mixed with the rest of the deoxygenated blood, coming from the bypass. For the 100% pulmonary shunt, the lung-oxy was not supplied with gas.
(3) During oxygenator support, an oxygenator (multiECCO2R, Euroset, Medolla, Italy) was connected in three different configurations: RA-PA, PA-LA, and Ao-LA. For a RA-PA configuration, part of the blood before the lung-bypass system was deviated into an oxygenator and introduced again in the system before the lung-bypass system. For PA-LA, part of the blood was deviated into an oxygenator before lung-bypass system and introduced again in the system after the lung-bypass system. For the Ao-LA configuration, the blood was withdrawn after passing through the lung-bypass system and returned to the left atrium. Considering that both connection sites are positioned after the lungs, the blood re-entry was located before the position where it was taken from (Supplemental Material A2).

For each configuration, three blood flow rates through the oxygenator support were investigated: 200, 300, and 400 mL/min. The arterial SO_2_ was measured at predefined intervals using a blood gas analyzer (ABL800 Flex, Radiometer, Denmark). Each experiment was repeated two times and a mean of the arterial SO_2_ values were calculated and plotted over time for each configuration and each percentage of pulmonary shunt. Given the complexity of the experiment, our main goal in comparing between the central configurations instead of having an absolute value of the steady state reached by any of them, and since the two repetitions brought the same results, the focus was more in reproducing the same exact boundary conditions for each configuration instead of reaching a high number of repetitions.

### Numerical simulation

A script in MATLAB (The MathWorks Inc., Natick, MA, USA) was created to simulate the pulmonary and systemic circulation. The simulation was performed for 50% and 100% pulmonary shunt, with an oxygenator connected in three different configurations to support the lungs. The initial conditions were determined in vitro. A more detailed explanation of the code is shown in Supplementary Material A1.

### Statistics

The data analysis, graphical presentations, and numerical simulations were performed using MATLAB.

## Results

1. In vitro simulation

**Figure 3.**
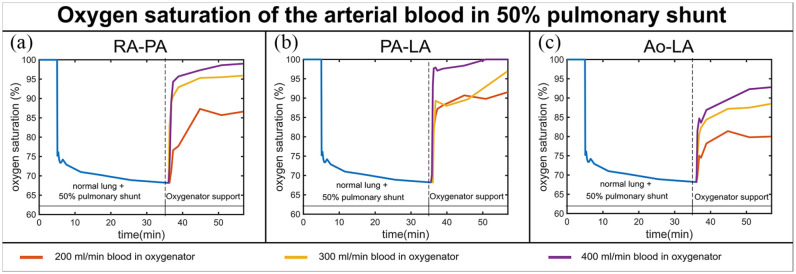
In vitro results of the SO_2_ time series for the three phases of the experiment (normal lung function, pulmonary shunt, and oxygenator support) for a 50% pulmonary shunt, three blood flows through the oxygenator (200, 300, 400 mL/min when the total blood flow in the system is 600 mL/min) and three different configurations: RA-PA (a), PA-LA (b), and Ao-LA (c).

**Figure 4.**
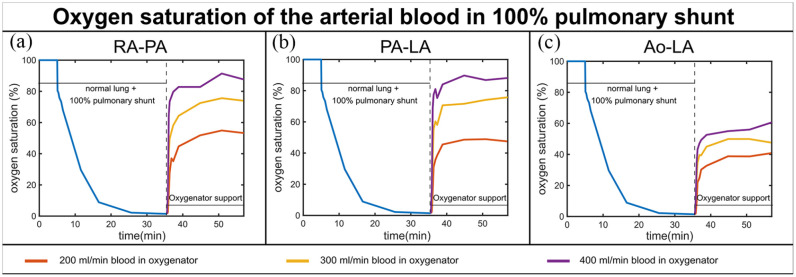
In vitro results of the SO_2_ time series for the three phases of the experiment (normal lung function, pulmonary shunt, and oxygenator support) for a 100% pulmonary shunt, three blood flows through the oxygenator (200, 300, 400 mL/min when the total blood flow in the system is 600 mL/min) and three different configurations: RA-PA (a), PA-LA (b), and Ao-LA (c).

The in vitro circulation was capable of simulating the oxygen exchange in a normal lung, pulmonary shunt, and during the oxygenator support. Figures 3 and 4 show the arterial SO_2_ over time for the three phases of the experiment.

1.1. 50% pulmonary shunt

During the 50% pulmonary shunt, arterial SO_2_ decreased from the physiological value of 100 to 68% nearly 20 min after the shunt was set. For RA-PA and PA-LA, physiological values were reached in case of a 50% pulmonary shunt, for both blood flow rates 300 and 400 mL/min. The RA-PA configuration had a comparable performance to PA-LA for both degrees of pulmonary shunt. Ao-LA could not reach physiological values of SO_2_, even with a pulmonary shunt of 50% and a blood flow through the oxygenator of 400 mL/min (Figure 3).

1.2 100% pulmonary shunt

In case of a 100% shunt, arterial SO_2_ decreased in 20 min from 100% to nearly 0%. SO_2_ increased when an oxygenator was connected in either configuration. The higher the blood flow through the oxygenator, the higher was the resulting SO_2_ value. The best SO_2_ value achieved was around 90% for RA-PA and PA-LA at 400 mL/min blood flow through the oxygenator; for the same conditions in Ao-LA configuration, the SO_2_ value reached was nearly 60% (Figure 4).

2. Numerical simulation

**Figure 5.**
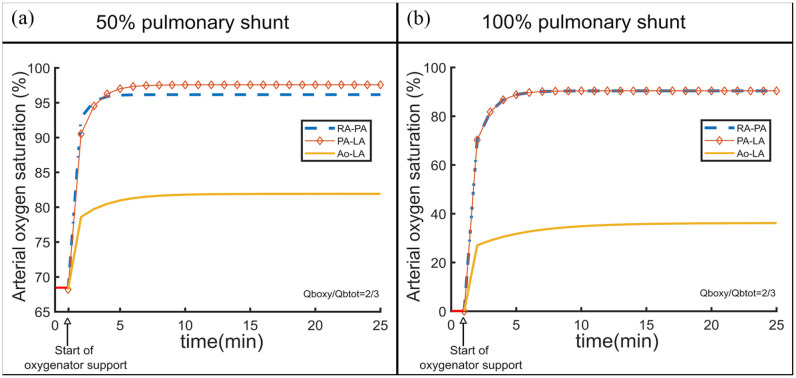
Numerically simulated time series of the arterial oxygen saturation for a 50% pulmonary shunt (a) and a 100% pulmonary shunt (b) when an oxygenator is connected in three different configurations (RA-PA, PA-LA, and Ao-LA) and the blood flow through the oxygenator is 2/3 of the total blood flow.

The numerical simulation shows the time series of the arterial SO_2_ for two percentages of pulmonary shunt (50% and 100%) and an oxygenator connected in three different configurations to support the lungs. The results of the numerical simulation, when the blood flow rate through the oxygenator is 2/3 of the total blood flow, are shown in Figure 5. The initial value of SO_2_ was measured in vitro as the steady state value reached for that percentage of pulmonary shunt.

2.1. 50% pulmonary shunt

For the 50% shunt, arterial SO_2_ trend started at an initial value of 68% and increased to 97% in case of PA-LA, a little bit above RA-PA, and was around 82% for Ao-LA. In this case, PA-LA had a slightly better performance than RA-PA (Figure 5(a)).

2.2. 100% pulmonary shunt

For a 100% shunt, the initial value of SO_2_ was near zero and as soon as an oxygenator was connected, SO_2_ started increasing until it reached a steady state. The steady state saturation achieved was around 90% in case of RA-PA and PA-LA and shortly below 40% in case of the Ao-LA configuration. The time series of SO_2_ for the configurations RA-PA and PA-LA is identical if the lungs do not function at all (Figure 5(b)) as that the oxygen content in PA is the same as the oxygen content in LA.

## Discussion

There are several possibilities of connecting a future central AL. Each connection influences gas exchanging properties as they depend on the perfusion route chosen. There is, however, no detailed information about the relation between these anatomically different connections and their physiological properties especially regarding gas exchange.

An in vitro circulation with a high number of components (four roller pumps, three oxygenators, and a reservoir) and a numerical simulation were able to simulate both systemic and pulmonary circulation for three different configurations. The results from the in vitro investigation show the time series of arterial SO_2_ from the moment the shunt was set till the moment the oxygenator support stabilizes SO_2_ values; the numerical simulation shows the arterial SO_2_ time series during the oxygenator support in three different configurations.

### RA-PA configuration

The RA-PA configuration represents a connection of the AL in series with the lungs, and it is similar to a current VV ECMO. The VV ECMO in the configuration inferior vena cava (IVC)-superior vena cava (SVC) does not influence the hemodynamics, as the amount of blood in RA drained by one cannula is directly replaced by the other.^1^ The RA-PA, however, drains the blood from the RA and returns it to the PA. The pressure difference from the RA to PA does not allow a passive flow but rather a pump. As a consequence of the return of the blood directly to the PA, the right ventricle is decompressed. Nevertheless, the amount of blood that has to be pumped through the pulmonary circulation stays the same and long term physiological consequences are unclear.

Regarding oxygenation, the performance of the RA-PA connection is similar to that of PA-LA for any percentage of pulmonary shunt; when the lung does not work at all, the two performances are mathematically identical.

### PA-LA configuration

Different studies suggest connecting an AL between the PA and LA for patients who require oxygenation and decarboxylation and suffer from right heart insufficiency as well.^10–12^ The biggest advantage of this configuration is the physiologic pressure difference between PA and LA, which allows, to a certain degree, a use without a pump. A pumpless device may facilitate an ambulatory support. Furthermore, it avoids high blood shear stresses of rotatory pumps.^2^ The difference in blood pressure between PA and LA allows passive blood flows up to 2.6 L/min as reported by Spillner et al.^8^ when a low-resistance membrane oxygenator (iLA Membrane Ventilator, Novalung^®^) is connected in the middle of the PA-LA shunt or 3.5 L/min in case of a paracorporeal artificial lung implanted between PA and LA.^13^ The iLA membrane ventilator, however, has a resistance of 5 mmHg/[L/min] to blood flow, which is five times that of a native lung.^7^ Therefore, new low-resistance gas exchangers are needed. The working group of Cook reported a new compliant thoracic artificial lung (cTAL) with an average blood resistance of 0.51 ± 0.03 mmHg/(L/min).^14^ The cTAL was used in a 14 days in vivo testing with sheep and the long term results showed no major thrombus formation and the ability to maintain normal physiology of the sheep.^15^

The reached blood flow rates with a low-resistance gas exchanger connected between PA and LA allow physiologically sufficient levels of oxygenation and CO_2_ elimination. Our in vitro and simulated data are consistent with these studies. As shown in Figure 3 in case of a 50% pulmonary shunt, this configuration can bring SO_2_ to physiological values even at blood flow rates that amount to half the total blood flow rate of the system.

The PA-LA configuration unloads the right ventricle as it returns the oxygenated blood directly to the left side of the heart, decompresses the pulmonary vasculature, and may lower mortality rate in patients with cardiogenic shock.^16^ The oxygenator connected in parallel to the lungs offers respiratory as well as circulatory support.^8,17^ However, PA-LA configuration requires surgery via thoracotomy and is therefore associated with the risks of surgical intervention.^18^

Furthermore, it introduces the possibility of a thrombus in the LA and as a consequence, runs the risk of systemic embolization.^19^

### Ao-LA configuration

Although a quite unusual and nearly neglected configuration, Ao-LA represents exemplarily an exclusively left sided passive shunt. There is a significant difference of blood pressure between the two sites, which allows high pumpless flow rates. The performance of this configuration, as shown from the in vitro results as well as simulation, is quite low compared to the other two: while RA-PA and PA-LA could reach physiological values of SO_2_ for a 50% pulmonary shunt and 400 mL/min blood flow through oxygenator, Ao-LA could only reach a maximum SO_2_ of nearly 60%. Nevertheless it has a big advantage: a high volume passive shunt may be instituted by this configuration. However, higher flows will lead to left atrial- and ventricular volume load, like an aortic valve insufficiency, and as a consequence, will limit its use as a high volume passive shunt.

### Study limitations

Both approaches present some limitations. Firstly, for the in vitro approach, four roller pumps and three oxygenators were used. The resulting circuit was very intricate and led quickly to hemolysis. To avoid biased data, the hemolysis was checked regularly during the experiment. Secondly, in vitro investigations were very long and the performance of the components of the circuit was affected by these long time periods. Furthermore, the results depend on the specific type of the oxygenator used. However, since the aim of this work was the comparison between the performance of an oxygenator in three different configurations, the same exact conditions (and especially the same specific oxygenator) were recreated throughout the experiment. Lastly, the mechanically composed circuit and the mathematical simulation could not fully represent the human body as they could not take into account the ventilation, the body’s reaction to low arterial SO_2_, compensation mechanisms, and the systemic need for oxygen. During in vitro investigations, OTR in the body periphery was limited by the deoxygenator’s performance, while for the mathematical simulation, it was considered a constant value (~300 mL/min) regardless of the incapacity of the lungs to satisfy it. Furthermore, the oxygen consumption only during rest was taken into account; in case of high intensity exercise, it may reach values as high as 5000 mL/min.^20^

In addition, the numerical simulation is based on saturation differences and does not take into account pO_2_ or the dependency of the saturation curve on pH. The simulation is not the most accurate and complete but gives a general overview of the capabilities of the setup.

Nevertheless, both in vitro and numerical simulations were able to characterize the physiological properties of possible, central anatomic configurations. For a more detailed assessment and comparison between the different configurations, further investigations also taking into account the CO_2_ transfer seem necessary to fully characterize the different configurations as a whole. This work focused on investigating central cannulations as highest flow rates can be achieved and large cannulas and connections can be used.^21,22^ Since a central cannulation requires surgery and is therefore associated with the risks of surgical intervention,^18^ multiple minimally invasive approaches are being investigated.^23,24^

### Conclusion

Different anatomical configurations of gas exchange devices such as a future AL’s lead to different oxygenation properties. Right to right and right to left configurations have better oxygenation performance than left to left ones.

In vitro and numerical investigations are two valid approaches for general assessment, understanding and comparison between possible connections of a future AL, especially when CO_2_ elimination is integrated. They could provide a total overview of the saturation time trend since low values of saturation (near 0%) and high percentages of shunt could be considered. Such values would be dangerous for the living organism during in vivo studies. Nevertheless, in vivo investigations are planned in the future to gain insight into the pathophysiology of the connections and to further validate the results from this study.

## Supplemental Material

sj-pdf-1-jao-10.1177_03913988231168163 – Supplemental material for Oxygenation performance assessment of an artificial lung in different central anatomic configurations Click here for additional data file. Supplemental material, sj-pdf-1-jao-10.1177_03913988231168163 for Oxygenation performance assessment of an artificial lung in different central anatomic configurations by Flutura Hima, Sebastian Kalverkamp, Ali Kashefi, Khosrow Mottaghy, Rachad Zayat, Lasse Strudthoff, Jan Spillner and Foivos Mouzakis in The International Journal of Artificial Organs
