# Supplementary material for: Oxygenation performance assessment of an artificial lung in different central anatomic configurations
Source: Int J Artif Organs. 2023 Apr 12;46(5):295–302. doi: 10.1177/03913988231168163 (PMC10160396; doi:10.1177/03913988231168163)
Supplement: sj-pdf-1-jao-10.1177_03913988231168163 – Supplemental material for Oxygenation performance assessment of an artificial lung in different central anatomic configurations [file sj-pdf-1-jao-10.1177_03913988231168163.pdf]

Additional Material

A1: Numerical Simulation

Assumptions made during numerical simulation:

1. Body´s need for oxygen was considered constant and exactly as the amount of the oxygen transfer rate than can bring the saturation from 100 % in the atrial blood to 70 % in the venous one.
2. The pulmonary shunt was modeled by mixing oxygenated blood (coming from a working lung) with non-oxygenated blood in the exact percentage of the shunt considered.
3. Compensation mechanisms were not considered.
4. The oxygenator could bring the oxygen saturation of the blood to 100%.
5. Since the oxygen partial pressure in the blood coming from the lungs ($pO_{2(lung)}$) cannot exceed 100 mmHg, the term $0.00314 * pO_{2(lung)}$ is very small and can be neglected from the calculation of the oxygen transfer rate (OTR_{lung}).

The simulation was performed as a “for” cycle in which the exact instance “i” begins when the blood flows though point IN of the circuit in figure A1. The need of the body in terms of oxygen was assumed constant and the pulmonary shunt was modeled by mixing oxygenated blood (coming from a working lung) with non-oxygenated blood in the exact percentage of the shunt considered.

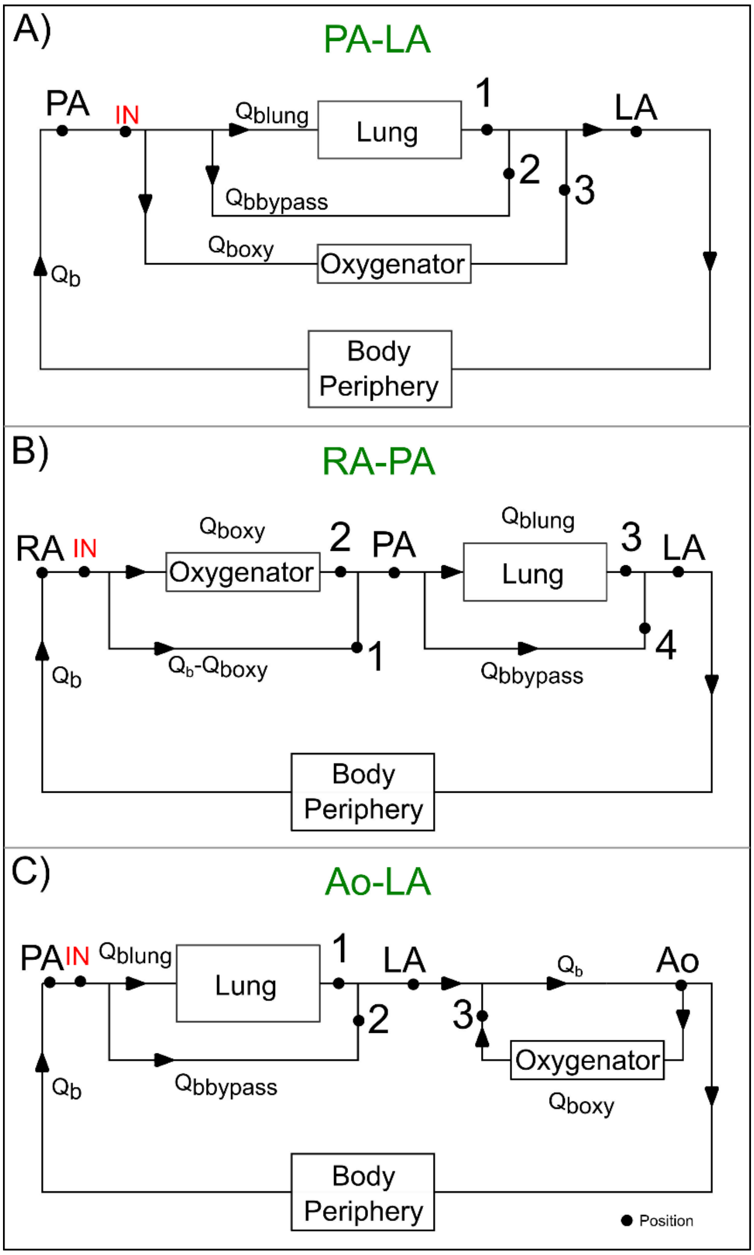

Figure A1:Block diagram of the three possible configurations: PA-LA (Figure A), RA-PA (Figure B), Ao-LA (Figure C).

Arterial saturation was calculated in 1 min intervals (each cycle) based on the venous saturation and the oxygen transfer rate of the lungs. The arterial and venous saturation were calculated in total for 25 cycles.

A commercially available oxygenator was tested in vitro, and the inlet/outlet blood-gas data were used to model the performance of a generic oxygenator. The contribution of the oxygenator was added according to the chosen configuration.

PA-LA

The blood from the PA flows through three ramifications: lung, bypass and oxygenator.

$$Q_b = Q_{blung} + Q_{bbypass} + Q_{boxy} \tag{1}$$

Where Q_b is the total blood flow in the system and Q_{blung}, $Q_{bbypass}$, and Q_{boxy} are the blood flows through the lungs, the bypass and the oxygenator respectively.

From the definition of the shunt:

$$Q_{bbypass} = d * \ Q_{blung} \tag{2}$$

where d is the percentage of the pulmonary shunt considered.

The oxygen transfer rate (OTR_{lung}) of the lungs was calculated as follows:

$$OTR_{lung} = (H * Hb * \Delta SO_2 + 0.00314 * pO_{2(lung)}) * Q_{blung}$$

where H=1,34 is a constant, Hb is the blood hemoglobin, ΔSO_2 is the difference between oxygen saturation of the arterial and venous blood, $pO_{2(lung)}$ is the oxygen partial pressure, and Q_{blung} is the blood flow rate through the lungs. Since the $pO_{2(lung)}$ cannot exceed 100 mmHg, the term $0.00314 * pO_{2(lung)}$ is very small and can be neglected.

$$OTR_{lung} = (H * Hb * \Delta SO_2) * Q_{blung} \tag{3}$$

We defined as normal transfer rate (OTR_{normal}) the oxygen transfer rate that by a total blood flow Q_b could increase the saturation from a venous value (SO_{2v}=70%) to an arterial value (SO_{2a}=100%).

$$OTR_{normal} = \left(H * Hb * (SO_{2a} - SO_{2v})\right) * Q_b \tag{4}$$

From equation (3) and (4):

$$OTR_{lung} = \frac{OTR_{normal}}{Q_b} * Q_{blung}$$

The oxygen content in LA ($V_{O2(LA)}$) can be calculated as the sum of the oxygen content in positions 1 ($V_{O2(1)}$), 2 ($V_{O2(2)}$), and 3 ($V_{O2(3)}$) of figure 1A each weighted by a factor:

$$V_{O2(LA)} = \left(V_{O2(1)} * Q_{blung} + V_{O2(2)} * Q_{bbypass} + V_{O2(3)} * Q_{boxy}\right)/Q_b \tag{5}$$

For position 1:

$$OTR_{lung} = (H * Hb * \left(SO_{2(1)} - SO_{2(PA)}\right)) * Q_{blung}$$

$SO_{2(1)}$: oxygen saturation in position 1

$SO_{2(PA)}$: oxygen saturation of the PA

$$SO_{2(1)} = SO_{2(PA)} + \frac{OTR_{lung}}{H * Hb * Q_{blung}} = SO_{2(PA)} + \frac{OTR_{normal}}{H * Hb * Q_b}$$

$$V_{O2(1)} = H * Hb * \frac{SO_{2(1)}}{100} \tag{6}$$

For position 2:

$$SO_{2(2)} = SO_{2(PA)}$$

$SO_{2(2)}$: oxygen saturation in position 2

$$V_{O2(2)} = H * Hb * \frac{SO_{2(2)}}{100} \tag{7}$$

For position 3:

The OTR of the oxygenator (OTR_{oxy}) was calculated as:

$$OTR_{oxy} = Q_{boxy} * (H * Hb * (SO_{2(3)} - SO_{2(PA)}) + 0.00314 * pO_{2(oxy)})$$

Where $pO_{2(oxy)}$ is the oxygen partial pressure of the blood coming from the oxygenator and $pO_{2(oxy)} = 680 * e^{(-0.21Q_{boxy}/100)}$ from in vitro data.

We assumed that the oxygenator can bring the oxygen saturation of the blood to 100%. Therefore $SO_{2(3)}$=100%

$SO_{2(3)}$: oxygen saturation in position 3

$$V_{O2(3)} = H * Hb * \frac{SO_{2(3)}}{100} + 0.00314 * pO_{2(oxy)} \tag{8}$$

Once calculating the oxygen content in LA, we can calculate the saturation as well:

$$SO_{2(LA)} = \frac{100 * V_{O2(LA)}}{H * Hb}$$

Once the blood flows through the body periphery, oxygen will be transferred to the body periphery and the oxygen saturation of the blood will change. As mentioned before, in a normal functioning lung, the saturation will change from 100% to approx. 70% (ΔSO_2=0,3). We consider for the simulation, a constant need of the body in terms of oxygen, so a constant OTR, and since OTR is directly proportional to the ΔSO_2, a constant ΔSO_2 of 0,3 too.

$$SO_{2(PA)} = SO_{2(LA)} - 0{,}3$$

RA-PA

The block diagram is shown in figure 1B.

For position 1:

$$SO_{2(1)} = SO_{2(RA)}$$

$SO_{2(1)}$: oxygen saturation in position 1

$SO_{2(RA)}$: oxygen saturation of the RA

$$V_{O2(1)} = H * Hb * \frac{SO_{2(1)}}{100}$$

For position 2:

The OTR of the oxygenator was calculated as:

$$OTR_{oxy} = Q_{boxy} * (H * Hb * (SO_{2(2)} - SO_{2(RA)}) + 0.00314 * pO_{2(oxy)})$$

where $pO_{2(oxy)} = 680 * e^{(-0.21 Q_{boxy}/100)}$ from in vitro data.

As before $SO_{2(2)}$=100%

$SO_{2(2)}$: oxygen saturation in position 2

$$V_{O2(2)} = H * Hb * \frac{SO_{2(2)}}{100} + 0.00314 * pO_{2(oxy)}$$

$V_{O2(PA)} = (V_{O2(1)} * (Q_b - Q_{boxy})$+ $V_{O2(2)} * Q_{boxy})$ / Q_b

$$SO_{2(PA)} = \frac{100 * V_{O2(PA)}}{H * Hb}$$

where $V_{O2(PA)}$ is the oxygen content in PA

$$Q_b = Q_{blung} + Q_{bbypass}$$

For position 3:

$$OTR_{lung} = (H * Hb * (SO_{2(3)} - SO_{2(PA)})) * Q_{blung}$$

$SO_{2(3)}$: oxygen saturation in position 3

$$SO_{2(3)} = SO_{2(PA)} + \frac{OTR_{lung}}{H * Hb * Q_{blung}} = SO_{2(PA)} + \frac{OTR_{normal}}{H * Hb * Q_b}$$

$$V_{O2(3)} = H * Hb * \frac{SO_{2(3)}}{100}$$

For position 4:

$$SO_{2(4)} = SO_{2(PA)}$$

$SO_{2(4)}$: oxygen saturation in position 4

$$V_{O2(4)} = H * Hb * \frac{SO_{2(4)}}{100}$$

$V_{O2(LA)} = (V_{O2(3)} * Q_{blung} + V_{O2(4)} * Q_{bbypass}) / Q_b$

$$SO_{2(LA)} = \frac{100 * V_{O2(LA)}}{H * Hb}$$

As explained for the first configuration:

$$SO_{2(RA)} = SO_{2(LA)} - 0{,}3$$

<u>Ao-LA</u>

The block diagram is shown in figure 1C.

$$Q_b = Q_{blung} + Q_{bbypass}$$

For position 1:

$$OTR_{lung} = (H * Hb * (SO_{2(1)} - SO_{2(PA)})) * Q_{blung}$$

$$SO_{2(1)} = SO_{2(PA)} + \frac{OTR_{lung}}{H * Hb * Q_{blung}} = SO_{2(PA)} + \frac{OTR_{normal}}{H * Hb * Q_b}$$

$$V_{O2(1)} = H * Hb * \frac{SO_{2(1)}}{100}$$

For position 2:

$$SO_{2(2)} = SO_{2(PA)}$$

$$V_{O2(2)} = H * Hb * \frac{SO_{2(2)}}{100}$$

For position 3:

The OTR of the oxygenator was calculated as:

$$OTR_{oxy} = Q_{boxy} * (H * Hb * (SO_{2(3)} - SO_{2(LA)}) + 0.00314 * pO_{2(oxy)})$$

where $pO_{2(oxy)} = 680 * e^{(-0.21Q_{boxy}/100)}$ from in vitro data and $SO_{2(3)}$=100%

$$V_{O2(3)} = H * Hb * \frac{SO_{2(3)}}{100} + 0.00314 * pO_{2(oxy)}$$

$V_{O2(LA)} = (V_{O2(1)} * Q_{blung} + V_{O2(2)} * Q_{bbypass} + V_{O2(3)} * Q_{boxy}) / (Q_b + Q_{boxy})$

$$SO_{2(LA)} = \frac{100 * V_{O2(LA)}}{H * Hb}$$

$$SO_{2(PA)} = SO_{2(LA)} - 0{,}3$$

A2: Schematic representation of the three possible central connections of a future artificial lung: RA-PA, PA-LA and Ao-LA

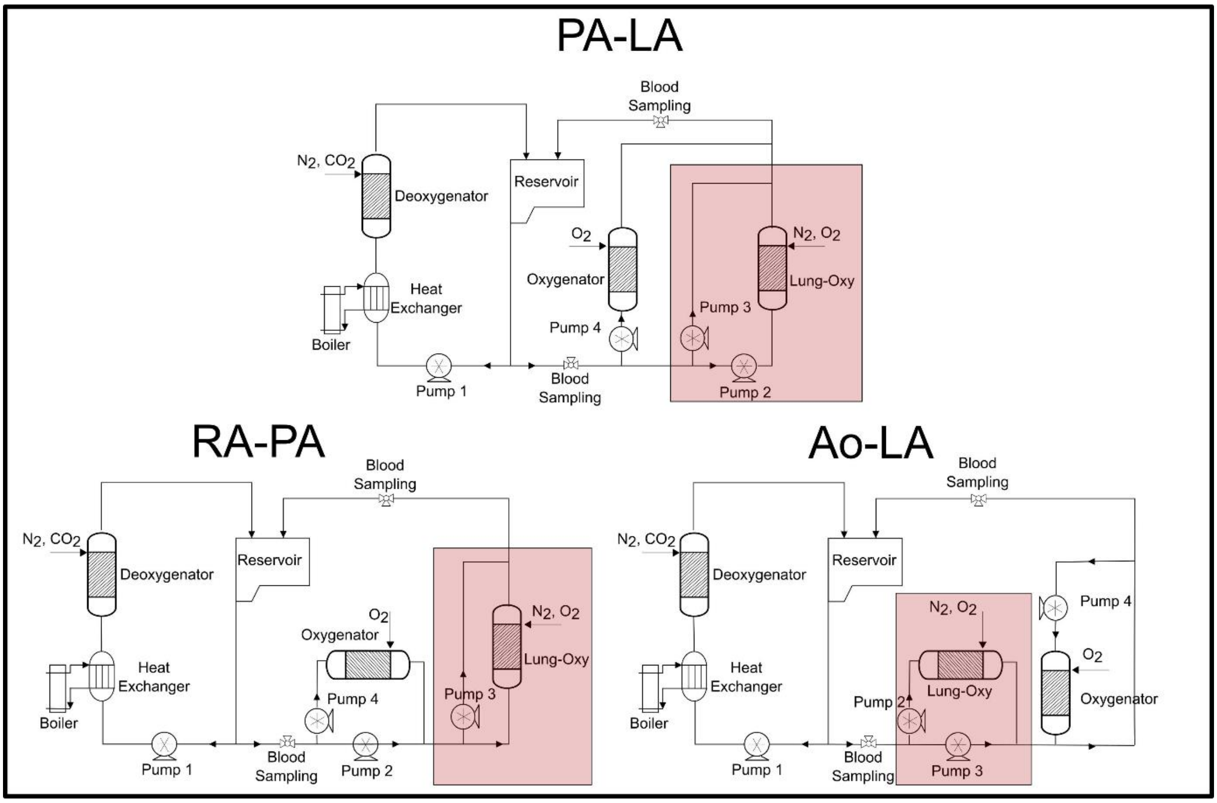

Figure A2: Schematic representation of the three possible central connections of a future artificial lung: RA -PA, PA-LA and Ao-LA. The pulmonary shunt was simulated by an oxygenator in parallel with a shunt (highlighted in red).
